# An analysis of respondent-driven sampling with injecting drug users in a high HIV prevalent state of India

**DOI:** 10.1186/s12954-017-0171-0

**Published:** 2017-07-03

**Authors:** Sanjib Kumar Phukan, Gajendra Kumar Medhi, Jagadish Mahanta, Rajatashuvra Adhikary, Gay Thongamba, Ramesh S. Paranjape, Brogen S. Akoijam

**Affiliations:** 1 Regional Medical Research Centre (RMRC), N.E. Region (ICMR), Post Box No. 105, Dibrugarh, AS 786 001 India; 2 Department of Community Medicine, North East Indira Gandhi Regional Institute of Health and Medical Science (NEIGRIHMS), Shillong, ML India; 3 UNAIDS India Country Office, 11 Olof Palme Marg, Vasant Vihar, New Delhi, 110 057 India; 4 FHI 360, H-5 (Ground Floor), Green Park Extension, New Delhi, 110 016 India; 5 National AIDS Research Institute (NARI), Plot No. 73, Block G, MIDC Complex, Bhosari, Pune 411 026 India; 6 Regional Institute of Medical Science (RIMS), Lamphelpat, Imphal, MN 795004 India

**Keywords:** Personal network, Younger IDUs, Sharing needle syringe, Respondent-driven sampling, HIV

## Abstract

**Background:**

Personal networks are significant social spaces to spread of HIV or other blood-borne infections among hard-to-reach population, viz., injecting drug users, female sex workers, etc. Sharing of infected needles or syringes among drug users is one of the major routes of HIV transmission in Manipur, a high HIV prevalence state in India. This study was carried out to describe the network characteristics and recruitment patterns of injecting drug users and to assess the association of personal network with injecting risky behaviors in Manipur.

**Methods:**

A total of 821 injecting drug users were recruited into the study using respondent-driven sampling (RDS) from Bishnupur and Churachandpur districts of Manipur; data on demographic characteristics, HIV risk behaviors, and network size were collected from them. Transition probability matrices and homophily indices were used to describe the network characteristics, and recruitment patterns of injecting drug users. Univariate and multivariate binary logistic regression models were performed to analyze the association between the personal networks and sharing of needles or syringes.

**Results:**

The average network size was similar in both the districts. Recruitment analysis indicates injecting drug users were mostly engaged in mixed age group setting for injecting practice. Ever married and new injectors showed lack of in-group ties. Younger injecting drug users had mainly recruited older injecting drug users from their personal network. In logistic regression analysis, higher personal network was found to be significantly associated with increased likelihood of injecting risky behaviors.

**Conclusion:**

Because of mixed personal network of new injectors and higher network density associated with HIV exposure, older injecting drug users may act as a link for HIV transmission or other blood-borne infections to new injectors and also to their sexual partners. The information from this study may be useful to understanding the network pattern of injecting drug users for enriching the HIV prevention in this region.

## Background

Accessing “hard-to-reach” populations such as injecting drug users (IDUs), female sex workers (FSWs), men who have sex with men (MSM), and clients of sex workers for research activities has historically been challenging due to the non-availability of their sampling frame and their stigmatized behavior [1]. These populations constantly try to avoid open contact with the rest of the society. The technique respondent-driven sampling (RDS), pioneered by sociologist Douglass D. Heckathorn in the mid of 1990s, is generally considered a methodology to access hard-to-reach populations through their personal or social network and can generate unbiased estimates of the prevalence of a disease risk factors or other characteristics in a socially networked population [1, 2].

Research on social network helps to study the social setting of drug users’ or other hard-to-reach populations’ health risks that are associated with the spread of human immunodeficiency virus (HIV), sexually transmitted infections, and other blood-borne diseases [3]. IDUs social network may play an important role in drug use practices. The risky behaviors that are related to HIV are embedded in dynamic social networks that connect individual to others through interaction [4]. The IDU members connected through social network to each other, like themselves, may influence to get involved the risky behavior of HIV or hepatitis C virus (HCV) that assist disease transmission [5–7]. Various network characteristics such as size of network, sharing injecting equipment [8–11], drug injection network size, and network dynamics [12, 13] have been found to be associated with HIV risk.

According to the National AIDS Control Organization (NACO), India, sharing of infected needle or syringe was the major route of HIV transmission in the northeastern region of India [14]. The prevalence of HIV recorded among IDU in Manipur was 12.1% which is much higher than the overall estimates (9.9%) of the country [15].

Social network of IDUs is a significant context to understanding the network pattern of drug users and its various intersections which may influence the spread of HIV infection. Using the RDS analysis tool (RDSAT), we can understand various network characteristics of IDUs that may increase their susceptibility for exposure to HIV and other blood-borne infections. The present study was conducted in Bishnupur and Churachandpur of Manipur, one of the highest HIV prevalence states in India. The objective of the present study was to assess the network characteristic and recruitment pattern of IDUs through transition probability matrices and homophily indices with respect to different characteristics, and to assess the association between personal networks and sharing of needle/syringe or other injecting equipment among IDUs in Manipur.

## Methods

This cross-sectional study was carried out among IDUs in the Bishnupur and Churachandpur districts of Manipur from 2009 to 2010, which is known as (Integrated Behavioral and Biological Assessment) IBBA Round-II. The IBBA data collection was approved by Protection of Human Subjects Committee (Family Health International, 360), Health Ministry Screening Committee (Indian Council of Medical Research), and the ethical committee of Regional Medical Research Centre (RMRC). The detail summary of the IBBA objectives, sampling methods used, and questionnaire are described elsewhere [16, 17].

IDUs in this region are a hard-to-reach group due to illegal nature of drug use and social stigma attached to it. In this study, RDS technique was used to recruit the participants. RDS is a chain-referral technique which is used for hidden population like IDUs, FSWs, MSM [1, 2] and which can overcome the limitations of other sampling methods to attain more representative sample from such hard-to-reach population [18]. RDS method allows in estimating asymptomatic unbiased estimator of population parameters extenuating the biases of chain-referral sampling using RDSAT [19]. In RDS, sampling process begins with few non-randomly selected initial recruits (called “seeds”) from the target population who meet the eligibility criteria. These seeds then start the chain referral by recruiting fixed numbers of eligible peers from their personal network who, in turn, recruit other peers for the study. This recruitment process continues until the target sample size is attained [18]. The target sample size for the study was calculated as 400 [17, 18]. In this study, we recruited four purposively selected seeds from the target population of each selected district who met the eligibility criteria to initiate the recruitment process. Each seeds were given uniquely coded coupons to recruit maximum of three eligible peers from their personal networks. This requirement process continued till the target sample size was achieved in the study. All the seeds circulated at least 7th waves in both the districts. Only about 8 weeks was required to recruit the required samples in the study. More detailed description of sampling design adopted to recruit participants in this study has been already described elsewhere [16–18].

To know the personal network size of the participants, we asked: “how many male IDUs do you personally know and they also know you?” The definition of an IDU was any man, 18 years or older, who has injected drugs for non-medical reason at least once in the last 6 months. After obtaining written informed consent, anonymous face-to-face interviews were conducted by trained interviewers who collected data using pre-coded, closed-ended questionnaires from the eligible respondents. The dependent variable was personal network size of the IDUs. Other variables of interest were socio demographic characteristics, duration of injecting drug used, and sharing of needle/syringe or other injecting equipment.

RDS population estimates with 95% confidence intervals (CI), transition probability matrices, and homophily indices can be calculated by RDSAT 7.1 [20].The assumption of RDS is that the network of study groups is connected and that every subject is reachable from every other subject, which may describe as a process of regular Markov chain [19]. In RDS, the transition probability matrix describes the probability of one group recruiting another group [21]. The homophily index (denoted by *H*) describes the extent of in-group ties. A homophily index, *H* = 1.0 reflects perfect homophily, indicating that all ties are formed with other members of the same group; and *H* = −1.0 reflects perfect heterophily, indicating that ties are formed completely outside of the group. Intermediate levels of homophily are defined in a parallel manner. A homophily of 0.12 (or <1) or a homophily of 12.0% means that the respondents form their networks as though 12.0% of the time they form a tie to another person like themselves, the rest of time they form ties through random mixing, that is, forming ties irrespective of groups membership. Accordingly, a homophily of −0.12 means that the respondents form their networks as though 12.0% of the time they form a tie to someone unlike themselves, and the rest of time form networks through random mixing, which reflects heterophily within the groups [2]. Univariate binary logistic regression was performed to calculate crude odds ratio (cOR), and multivariate logistic regression was performed to calculate adjusted odds ratio (aOR) with corresponding 95% CI by using SPSS for determining independent association between personal network size and sharing of needle/syringe or other injecting equipment by adjusting potential factors, viz., current age, educational status, marital status, and duration of injection.

## Results

A total of 821 IDUs, 410 from Bishnupur and 411 from Churachandpur, were sampled in the study. The average age of the participants was 26 and 29 years in Bishnupur and Churachandpur respectively. Out of all IDUs, 47.9 and 26.1% completed 10th standard, 61.0 and 43.7% were never married, and 42.6 and 53.5% had 6 years and more history of injecting drug in Bishnupur and Churachandpur respectively (Table 1).

**Table 1:** Characteristics of IDU participants

Category	Bishnupur	Churachandpur
Current age		
18–24	28.6 (23.6–33.6)	16.0 (12.3–19.9)
25–30	39.7 (34.8–44.5)	44.0 (39.3–48.6)
>30	31.7 (27.3–36.5)	40.0 (35.1–45.1)
Highest grade completed		
Illiterate	6.2 (4.0–8.6)	4.2 (2.3–6.5)
≤10th standard	45.9 (40.9–50.6)	69.7 (65.0–74.3)
>10th standard	47.9 (43.1–52.9)	26.1 (21.7–30.5)
Marital status		
Currently married	34.6 (29.5–39.7)	36.7 (32.1–42.2)
Ever married^a^	4.4 (2.9–6.2)	19.6 (15.7–23.5)
Never married	61.0 (55.5–66.1)	43.7 (38.2–48.3)
Duration of injecting drug		
<1 year	9.5 (6.9–12.9)	2.4 (1.0–4.2)
1–2 years	21.9 (17.4–25.9)	13.7 (10.3–17.2)
3–5 years	26.0 (22.3–30.8)	30.4 (25.6–34.7)
6 years and more	42.6 (37.1–47.2)	53.5 (48.8–58.8)

The numbers in parentheses represent 95% CI calculated by RDSAT 7.1

^a^Separated and widowed IDUs

Almost 20.0% of IDUs had more than 10 members in their personal networks; and at least half of the total IDUs reported that they had up to 5 members in their network in both the districts. Nearly 30.0 and 17.0% IDUs reported that they had 6–10 members in their personal network in Bishnupur and Churachandpur respectively (data not shown).

In this study, the size of personal network was determined by asking the respondents—“how many male IDUs do you personally know and they also know you?” Average network size of IDU’s was similar (8.8 and 8.1) in both the districts. Table 2 represents the recruitment pattern of IDUs in the Bishnupur and Churachandpur districts. Affiliation pattern of IDUs age showed a mixed age group setting in both the districts. Up to 14.0% of times, IDUs form a tie to another person’s like themselves, and rest of the time, they form ties through random mixing from irrespective of group membership.

**Table 2:** Transition probability matrices and homophily indices of different factors of IDUs using RDSAT 7.1

Recruiters	Recruitees							
	Bishnupur [figure in %]				Churachandpur [figure in %]			
	18–24	25–30	≥31		18–24	25–30	≥31	
Age								
18–24	29.6	40.8	29.6		20.0	47.3	32.7	
25–30	22.5	42.7	34.8		16.8	44.7	38.5	
≥31	15.2	43.5	41.3		13.0	46.9	46.9	
Homophily	0.015	0.051	0.140		0.047	0.014	0.002	
	Illiterate	≤10th	>10th		Illiterate	≤10th	>10th	
Educational status								
Illiterate	5.0	55.0	40.0		13.3	53.3	33.4	
≤10th	7.0	47.7	45.3		3.9	70.2	25.9	
>10th	6.6	39.4	54.0		3.0	67.2	29.8	
Homophily	−0.185	0.032	0.117		0.096	0.017	0.050	
	Currently married	Ever married	Never married		Currently married	Ever married	Never married	
Marital status								
Currently married	47.8	6.7	45.5		39.6	19.4	41.0	
Ever married	52.6	0.0	47.4		34.7	13.3	52.0	
Never married	31.1	5.9	63.0		33.8	17.7	48.5	
Homophily	0.203	−1.0	0.053		0.045	−0.319	0.084	
	<1 year	1–2 years	3–5 years	6 years and more	<1 year	1–2 years	3–5 years	6 years and more
Duration of injecting drug								
<1 year	2.6	10.5	26.3	60.6	0.0	14.3	14.3	71.4
1–2 years	7.0	19.3	33.3	40.4	2.1	18.8	35.4	43.7
3–5 years	8.4	21.1	24.2	46.3	1.9	12.6	32.0	53.5
6 years and more	7.4	18.6	23.3	50.7	2.0	14.6	27.2	56.2
Homophily	−0.722	−0.118	−0.070	0.141	−1.0	0.059	0.024	0.057

In Bishnupur, affiliation pattern of educational status reflected a trend of heterophily (*H* = −0.185) in illiterate IDUs. IDUs who were reported as illiterate, recruited only 5.0% (*H* = −0.185) and 13.3% (*H* = 0.096) of the time like themselves to form their network and rest of the time (33.3 to 55.0% of the time) they formed their network through random mixing. Result also showed less interaction from literate (≤10th and >10th standard) to illiterate IDUs, only 3.0 to 7.0% of the time literate IDUs recruited illiterate IDUs from their personal network in both the districts; this is probably due to small number of illiterate IDUs recruited in the study.

The affiliation indices of ever married and new injectors (<1 year of injecting drug) reflect complete heterophily (*H* = −1.0) which indicates a lack of in-group ties in both the districts. Near about 50.0% of the time currently married IDUs in Bishnupur and never married IDUs in Churachandpur recruited other IDU like themselves and remaining of the time they formed their network through random mixing. The affiliation pattern of duration of injecting drugs also indicated a strong heterophily (*H* = −0.722) among new injectors in Bishnupur. In Bishnupur and Churachandpur, new IDU had mainly recruited older IDUs who had 6 years or more history of injecting practices 60.0 and 71.4% of the time from their personal network respectively. The homophily indices were −0.118 and 0.059 for 1–2 years, −0.070 and 0.024 for 3–5 years, and 0.141 and 0.057 for 6 years and more injecting duration of IDUs in Bishnupur and Churachandpur respectively.

In univariate analysis, higher personal network was significantly associated with increased risk of sharing needle syringe or other injecting equipment in both the districts [cOR 1.78, CI 1.03–3.05, *p*-value 0.038 in Bishnupur and cOR 2.30, CI 1.28–4.14, *p*-value 0.005 in Churachandpur]. Multivariate binary logistic regression was performed to assess the independent association between personal network size and sharing risky behaviors by adjusting current age, educational status, marital status, and duration of injection in the model. In Churachandpur, higher personal network size was found to be significantly associated with increased likelihood of sharing needle syringe or drug solutions or other injecting equipment [aOR 2.37, CI 1.31–4.28, *p*-value 0.004] and also found existence of a similar trend [aOR 1.67, CI 0.95–2.93, *p*-value 0.076] in the Bishnupur district. Though statistically not significant, increasing personal networks showed the increased possibility of sharing needle/syringe or other injecting equipment in both the districts (Table 3).

**Table 3:** Binary logistic regression for injecting risky behaviors across the personal network size

	Shared needle syringe or drug solutions or other injecting equipments			
	Bishnupur		Churachandpur	
	cOR (95% CI)	aOR^a^ (95% CI)	cOR (95% CI)	aOR^a^ (95% CI)
Personal network size				
Up to 5	Reference	Reference	Reference	Reference
6–10	1.41 (0.90–2.19)	1.34 (0.85–2.12)	1.01 (0.57–1.76)	1.03 (0.58–1.81)
11 and more	1.78 (1.03–3.05)^*^	1.67 (0.95–2.93)^†^	2.30 (1.28–4.14)^**^	2.37 (1.31–4.28)^**^

^*^ *p* < 0.05, ^**^ *p* < 0.01, ^†^borderline significant

^a^Adjusted for current age, educational status, marital status, duration of injection

## Discussion

To the best of our knowledge, this study was the first study to assess the personal network characteristics of IDUs in the state of Manipur using RDS. Personal networks play an important role in influencing HIV risk behaviors. In Bishnupur, the samples were relatively younger than the Churachandpur samples. The younger age of IDUs in Bishnupur indicates a longer duration of injecting practices which may allow more risky injecting practices and greater chance of HIV transmission or other blood-borne infections.

Recruitment analysis showed that the recruitment patterns of IDUs with respect to different age groups were similar in both the districts. Homophily indices reflected that there was no strong tie in Manipur within the same age group of IDUs. It suggested that IDUs were mostly engaged in injecting practice in mixed age setting.

In Churachandpur, most of the illiterate IDUs appeared to be recruited by their own group. Transition probability matrix suggested that literate IDUs seemed to be dissociative and interacted less with illiterate IDUs. In contrast, in Bishnupur, illiterate IDUs were primarily recruited by literate IDUs.

Analysis suggested that in Bishnupur, IDUs formed a socially distinct group by marital status. Ever married IDUs did not seem to interact with another IDU like them. In Churachandpur also, ever married IDUs showed less interactions with another IDU like themselves. Result suggested that ever married IDUs were mostly recruited into the study by never married and currently married IDUs, which indicated that ever married IDUs mostly interacted with never married and currently married IDUs to engage in intravenous injecting practice. All the new IDUs (<1 year of duration) appeared to be recruited for the study by older IDUs who had more than 1 year history of injecting practices. Results showed a strong personal networking from new IDUs to older IDUs. It may suggest that IDU with lower duration of injecting practices were more likely to interact with IDUs who had higher injecting duration to build their personal network. Homophily index of new IDUs also showed complete heterophily in Churachandpur and strong heterophily in the Bishnupur district. This may suggest that new IDUs have been initiated into injecting practices by older IDUs. One possible explanation for the relationship from new to older IDUs in the context of Manipur may be due to their less experience in accessing drug markets and may be they have to rely on experienced IDUs to prepare the drug substance and to inject drugs. The relationship between new and older IDUs may be a significant cause to new IDUs for getting infected with HIV [22], as IDUs with higher duration of injecting practices are vulnerable to getting infected with HIV [23]. In our study, we found an increasing trend of prevalence of HIV (in Bishnupur 3.3% and in Churachandpur 12.5%) among new IDUs in both the districts.

In this study, we found, IDUs with higher personal network was significantly associated with sharing of needle syringe or other injecting equipment in both the districts. The significant association between higher personal network size of IDUs and injecting risky behavior; and the interaction of new IDUs with older IDUs may be a significant root for new IDUs to getting infected with HIV or other blood-borne infections. In our study, we found the presence of linear trend between personal network size and the duration of injecting drug use of IDUs (data not shown). Larger network size of IDUs showed more vulnerability of infected with HIV and HCV [24]. Previous study reported that large personal network of IDUs was significantly associated with greater frequency of injecting drug [25]. In our analysis, we also found that higher personal network size was significantly associated with increased risk of HIV/HCV seropositivity and frequency of drug injection (data not shown).

The basic limitation of our study is its cross-sectional design. In this study, only a limited number of personal networking questions were included. We also did not involve specific questions regarding whether there was any female injecting partner or family members involved in their personal network. Therefore, we are unable to describe the personal network pattern of IDUs in detail.

## Conclusion

The findings of this IDUs personal network study in this high HIV prevalence state of India indicate that new IDUs were more likely to interact with older IDUs in the context of drug injecting practices that may make them more vulnerable to HIV and other blood-borne infection through their interaction with older IDUs as prevalence of HIV or other blood borne infection is higher among older IDUs. Another notable finding of the study, which may have public health implication, is the association between higher network density and risky syringe sharing and HIV vulnerability. The findings of this report will be helpful to understand the social circumstance of HIV or other blood-borne infection among IDUs especially among new IDUs and may helpful to understand the HIV transmission dynamics and to enriching the HIV prevention in this region.

## Abbreviations

aOR: Adjusted odds ration
CI: Confidence interval
cOR: Crude odds ratio
FSW: Female sex worker
HCV: Hepatitis C virus
HIV: Human immunodeficiency virus
IBBA: Integrated behavioral and biological assessment
IDU: Injecting drug user
MSM: Men who have sex with men
RDS: Respondent-driven sampling
RDSAT: Respondent-driven sampling analysis tool

## References

[1] Heckathorn DD (1997). Respondent-driven sampling: a new approach to the study of hidden populations. Soc Probl.

[2] Heckathorn DD (2002). Respondent-driven sampling II: deriving valid population estimates from chain-referral samples of hidden populations. Soc Probl.

[3] Weeks MR, Clair S, Borgatti SP, Radda K, Schensul JJ (2002). Social networks of drug users in high-risk sites: finding the connections. AIDS Behav.

[4] Latkin CA, Davey-Rothwell MA, Knowlton AR, Alexander KA, Williams CT, Boodram B (2013). Social network approaches to recruitment, HIV prevention, medical care, and medication adherence. Journal of acquired immune deficiency syndromes (1999).

[5] Friedman SR, Neaigus A, Jose B, Curtis R, Goldstein M, Ildefonso G, … & Des Jarlais DC. Sociometric risk networks and risk for HIV infection. American Journal of Public Health. (1997;87(8):1289-1296. 10.2105/ajph.87.8.1289 PMC1381088 9279263

[6] Périssé ARS, Nery JADC (2007). The relevance of social network analysis on the epidemiology and prevention of sexually transmitted diseases. Cadernos de Saúde Pública.

[7] Parker M, Ward H, Day S (1998). Sexual networks and the transmission of HIV in London. J Biosoc Sci.

[8] Lakon CM, Ennett ST, Norton EC (2006). Mechanisms through which drug, sex partner, and friendship network characteristics relate to risky needle use among high risk youth and young adults. Soc Sci Med.

[9] Bohnert AS, Bradshaw CP, Latkin CA (2009). A social network perspective on heroin and cocaine use among adults: evidence of bidirectional influences. Addiction.

[10] Neaigus A, Friedman SR, Jose B, Goldstein MF, Curtis R, Ildefonso G, Des Jarlais DC (1996). High-risk personal networks and syringe sharing as risk factors for HIV infection among new drug injectors. J Acquir Immune Defic Syndr.

[11] Latkin CA, Kuramoto SJ, Davey-Rothwell MA, Tobin KE (2010). Social norms, social networks, and HIV risk behavior among injection drug users. AIDS Behav.

[12] Hoffmann JP, Su SS, Pach A (1997). Changes in network characteristics and HIV risk behavior among injection drug users. Drug Alcohol Depend.

[13] Suh T, Mandell W, Latkin C, Kim J (1997). Social network characteristics and injecting HIV-risk behaviors among street injection drug users. Drug Alcohol Depend.

[14] National AIDS Control Organization (NACO). Department of Aids Control. Ministry of Health & Family Welfare. India Annual Report 2013-14. Retrieved from: http://www.naco.gov.in/sites/default/files/NACO_English%202013-14.pdf. Accessed 4 Apr 2015.

[15] National AIDS Control Organization. National Integrated Biological and Behavioural Surveillance (IBBS), India 2014-15. New Delhi: NACO, Ministry of Health and Family Welfare, Govt. of India. 2015. Retrieved from:http://www.aidsdatahub.org/sites/default/files/highlight-reference/document/India_IBBS_report_2014-15.pdf.

[16] Chandrasekaran P, Dallabetta G, Loo V, Mills S, Saidel T, Adhikary R, Alary M, Lowndes CM, Boily M, Moore J (2008). Evaluation design for large-scale HIV prevention programmes: the case of Avahan, the India AIDS initiative. Aids.

[17] Saidel T, Adhikary R, Mainkar M, Dale J, Loo V, Rahman M, Ramesh BM, Paranjape RS (2008). Baseline integrated behavioural and biological assessment among most at-risk populations in six high-prevalence states of India: design and implementation challenges. Aids.

[18] Medhi GK, Mahanta J, Paranjape RS, Adhikary R, Laskar N, Ngully P (2012). Factors associated with HIV among female sex workers in a high HIV prevalent state of India. AIDS Care.

[19] Salganik MJ, Heckathorn DD (2004). Sampling and estimation in hidden populations using respondent‐driven sampling. Sociol Methodol.

[20] Volz E, Wejnert C, Cameron C, Spiller M, Barash V, Degani I, Heckathorn DD (2012). Respondent driven sampling analysis tool (RDSAT) version 7.1.

[21] Spiller M, Cameron C, & Heckathorn D. RDSAT 7.1 user manual. Cornell University; 2012. Retrieved from: http://respondentdrivensampling.com. Accessed 27 Mar 2015.

[22] Goldsamt LA, Harocopos A, Kobrak P, Jost JJ, Clatts MC (2010). Circumstances, pedagogy and rationales for injection initiation among new drug injectors. J Community Health.

[23] Eicher AD, Crofts N, Benjamin S, Deutschmann P, Rodger AJ (2000). A certain fate: spread of HIV among young injecting drug users in Manipur, north-east India. AIDS Care.

[24] Wylie J, Shah L, Jolly A (2006). Demographic, risk behaviour and personal network variables associated with prevalent hepatitis C, hepatitis B, and HIV infection in injection drug users in Winnipeg, Canada. BMC Public Health.

[25] Latkin C, Mandell W, Vlahov D, Knowlton A, Oziemkowska M, Celentano D (1995). Personal network characteristics as antecedents to needle-sharing and shooting gallery attendance. Soc Networks.

